# P-1828. Virulence-Associated Genes of Carbapenem-Resistant *Klebsiella pneumoniae* isolates in a National Government Referral Center in the Philippines

**DOI:** 10.1093/ofid/ofae631.1991

**Published:** 2025-01-29

**Authors:** Christian Francisco, Aurora Ines Aguilar, Angelo dela Tonga, Stessi Geganzo, Karla Kristel A Banares, Windell L Rivera, Sohei Harada, Yohei Doi, Yohei Doi

**Affiliations:** National Institutes of Health, University of the Philippines Manila, Manila, National Capital Region, Philippines; National Institutes of Health, University of the Philippines Manila, Manila, National Capital Region, Philippines; National Institutes of Health, University of the Philippines Manila, Manila, National Capital Region, Philippines; National Institutes of Health, University of the Philippines Manila, Manila, National Capital Region, Philippines; University of the Philippines - Philippine General Hospital, Manila, National Capital Region, Philippines; University of the Philippines Diliman, Quezon City, National Capital Region, Philippines; Toho University School of Medicine, Ota-ku, Tokyo, Japan; Fujita Health University, Aichi, Aichi, Japan; Fujita Health University, Aichi, Aichi, Japan

## Abstract

**Background:**

*Klebsiella pneumoniae* (KP) infections are common in the Philippines. Local carbapenem resistance (CR) is at 11-50%. In addition to the rising antimicrobial resistance (AMR), some types of KP cause disseminated infection that can lead to poor clinical outcomes. Aside from describing AMR, it is also important to identify virulence genes to fully understand the pathogenesis of KP infections. There is no local data on the characterization of virulence genes on KP isolates and its impact on patient outcomes.

**Methods:**

This is a cross-sectional study that utilized bio-banked CR KP isolates collected from November 2019 to January 2020 at the Philippine General Hospital, the national government referral center. Identity of the banked presumptive isolates were confirmed using polymerase chain reaction (PCR). AMR profile was investigated using VITEK MS (bioMérieux, USA). Multiplex PCR was employed to detect the following virulence genes: siderophore (*ybtS, entB, iutA, iucA*), fimbriae (*mrkD, fimH*), capsular polysaccharide synthesis (*rmpA*) and hypermucoviscosity (*magA*).

**Results:**

Preliminary analysis included 50 bio-banked isolates from patients with CR KP infections. Majority were from male (28, 56%) with a median age of 53 years. Most common co-morbidities include genitourinary malignancy (6, 12%) and diabetes mellitus (5, 10%). The majority of isolates were from the respiratory tract (21, 42%), followed by blood (10, 20%) and urine (8, 16%). Most infections were hospital acquired (43, 86%). Resistance to colistin was at 2%. Analysis of the virulence genes were conducted in 35 isolates. All isolates (35/35, 100%) tested positive for *entB*. Most isolates tested positive for *mrkD* (33/34, 97.1%) and *fimH* (33/34, 97.1%), followed by *ybtS* (25/34, 73.5%), *magA* (25/35, 71.4%) and *iutA* (4/18, 22.2%). None of the isolates tested positive for *rmpA*. Our study reported high mortality rate of 32%.

**Conclusion:**

Mortality is high with CR KP infections. Virulence genes were detected from the collected isolates but the impact of each gene of gene combinations to patient outcomes need to be investigated. This study supports the need for further research on AMR and hypervirulence of KP infections.

**Disclosures:**

**Aurora Ines Aguilar, MS (cand.)**, Manila HealthTek, Inc.: Part-time employee focused on developing educational tools for junior and senior high school students from the STEM strand **Sohei Harada, MD, PhD**, MSD: Honoraria|Shionogi: Honoraria **Yohei Doi, MD, PhD**, bioMerieux: Lecture fees|Entasis: Grant/Research Support|Fujifilm: Advisor/Consultant|Gilead Sciences: Advisor/Consultant|GSK: Advisor/Consultant|KANTO CHEMICAL CO.,INC.: Grant/Research Support|KANTO CHEMICAL CO.,INC.: Patent for genotyping kit|MeijiSeika Pharma: Advisor/Consultant|Moderna: Advisor/Consultant|MSD: Lecture fees|Pfizer: Advisor/Consultant|Shionogi & Co., Ltd.: Grant/Research Support|Shionogi & Co., Ltd.: Lecture fees

